# Mutations in Mismatch Repair Genes and Microsatellite Instability Status in Pancreatic Cancer

**DOI:** 10.3390/cancers16112111

**Published:** 2024-05-31

**Authors:** Marina Emelyanova, Anna Ikonnikova, Alexander Pushkov, Elena Pudova, George Krasnov, Anna Popova, Ilya Zhanin, Darya Khomich, Ivan Abramov, Sergei Tjulandin, Dmitry Gryadunov, Ilya Pokataev

**Affiliations:** 1 Center for Precision Genome Editing and Genetic Technologies for Biomedicine, Engelhardt Institute of Molecular Biology, Russian Academy of Sciences, Moscow 119991, Russia; anyuik@gmail.com (A.I.); pudova_elena@inbox.ru (E.P.); gskrasnov@mail.ru (G.K.); siferosu@gmail.com (D.K.); abriv@bk.ru (I.A.); gryadunov@gmail.com (D.G.); 2 Federal State Autonomous Institution “National Medical Research Center for Children’s Health” of the Ministry of Health of the Russian Federation, Moscow 119991, Russia; pushkovgenetika@gmail.com (A.P.); ilya_zhanin@outlook.com (I.Z.); 3 N.N. Blokhin National Medical Research Center for Oncology, Ministry of Health of the Russian Federation, Moscow 115522, Russia; annpopova93@gmail.com (A.P.); stjulandin@mail.ru (S.T.); pokia@mail.ru (I.P.); 4 City Clinical Cancer Hospital No 1, Moscow Department of Health, Moscow 129090, Russia

**Keywords:** pancreatic cancer, mismatch repair deficiency, microsatellite instability, *MLH1*, *MSH2*, *MSH6*, NGS

## Abstract

**Simple Summary:**

Immunotherapy may be beneficial for pancreatic cancer (PC) patients with mismatch repair (MMR) deficiency. Although it is known that MMR deficiency (MMR-D) can result from mutations in MMR genes, the prevalence of these mutations and their impact on the formation of the MMR-D phenotype in PC have been insufficiently researched. Microsatellite instability (MSI) is a hallmark of MMR-D. Here, we estimated the frequency of germline and somatic mutations in the three MMR genes (MLH1, MSH2, and MSH6) in PC patients, estimated the prevalence of MSI, and assessed the relationship between MMR genes mutations and MSI status in PC. In our study of an unselected cohort of PC patients, we identified germline and somatic alterations in MMR genes, however these alterations did not contribute to the MMR-D phenotype. Our findings underscore the necessity of evaluating tumor MMR-D status in PC patients with confirmed Lynch syndrome when deciding whether to administer immunotherapy.

**Abstract:**

Patients with pancreatic cancer (PC) showing mismatch repair (MMR) deficiency may benefit from immunotherapy. Microsatellite instability (MSI) is a hallmark of MMR deficiency (MMR-D). Here, we estimated the prevalence of MSI in PC, investigated germline and somatic mutations in the three MMR genes (*MLH1*, *MSH2*, and *MSH6*), and assessed the relationship between MMR genes mutations and MSI status in PC. Clinical specimens from PC patients were analyzed using targeted next-generation sequencing, including paired normal and tumor specimens from 155 patients, tumor-only specimens from 86 patients, and normal-only specimens from 379 patients. The MSI status of 235 PCs was assessed via PCR. Pathogenic/likely pathogenic (P/LP) germline variants in the MMR genes were identified in 1.1% of patients, while somatic variants were found in 2.6% of patients. No MSI-H tumors were detected. One patient carried two variants (P (VAF = 0.57) and LP (VAF = 0.25)) simultaneously; however, their germline/somatic status remains unknown due to the investigation focusing solely on the tumor and MSI analysis was not performed for this patient. MSI is rare in PC, even in tumors with MMR genes mutations. Our findings underscore the importance of assessing tumor MMR-D status in PC patients with confirmed Lynch syndrome when deciding whether to prescribe immunotherapy.

## 1. Introduction

Pancreatic cancer (PC) is one of the most aggressive types of cancer. Approximately 90% of cases of PC are pancreatic ductal adenocarcinoma [1]. The majority of patients with PC progress asymptomatically to either locally advanced or metastatic disease. This fact leads to surgical excision being only possible for 15–20% of patients [2]. Chemotherapy remains the mainstay of systemic treatment for patients with advanced PC [3]. PC is associated with an extremely poor prognosis. For all stages combined, the 5-year relative survival rate is 9% [4]. As such, there is a need to improve treatment options for PC.

Immune checkpoint inhibitors (ICIs) are a novel class of immunotherapy drugs that have made breakthroughs in the treatment of various solid tumors [5], significantly improving the survival rate of cancer patients [6,7]. Although ICIs have not shown sufficient efficacy in unselected cohorts of patients with PC [8,9], there is evidence of benefit for patients with mismatch repair (MMR) deficiency PC [10,11].

MMR deficiency (MMR-D) is characterized by the loss of function of the MMR pathway. It can be caused by mutations in one of the major MMR genes (*MLH1*, *MSH2*, *MSH6*, or *PMS2*) or their epigenetic silencing [12,13,14,15,16]. The failure of MMR results in an increase in unrepaired replicative errors, including in microsatellites [17,18]. Microsatellite instability (MSI) is one of the hallmarks of a defective MMR system [18], and can be evaluated using PCR or next-generation sequencing (NGS) [19,20,21]. Another phenotypic manifestation of MMR-D is loss of MMR protein expression (l-MMR), evaluated using immunohistochemistry (ICH) [22]. The concordance rate between MSI-PCR and MMR IHC testing is reported to be greater than 95% [23,24,25,26], and the MSI-NGS assay has similar concordance with MSI-PCR and MMR IHC [21].

Existing data on the prevalence of MMR-D in PC, adjudicated through MSI testing and IHC, are limited and contradictory; reported rates range widely from 0% to 75% due to different detection methods and study cohorts [10,19,27,28,29,30,31,32,33,34]. It is necessary to determine the frequency of MMR-D in PC and identify the factors that affect it.

Although it is known that MMR-D can result from mutations in MMR genes, the prevalence of these mutations and their impact on the formation of the MMR-D phenotype in PC have been insufficiently researched. In our study, we estimated the frequency of germline and somatic mutations in the *MLH1*, *MSH2*, and *MSH6* genes in PC patients using NGS. We then performed an MSI-PCR analysis for patients with available tumor tissue. Finally, we assessed the relationship between mutations in the *MLH1*, *MSH2*, and *MSH6* genes and MSI status in PC.

## 2. Materials and Methods

### 2.1. Patients and Samples

This study included 620 patients with PC who underwent treatment at the N.N. Blokhin National Medical Research Center for Oncology, Ministry of Health of the Russian Federation, from 2001 to 2021. The current study conformed to the principles of the Declaration of Helsinki.

The matched tumor and normal specimens were evaluated for 155 patients, tumor-only specimens were evaluated for 86 patients, and normal-only specimens (358 blood and 21 FFPE samples) were evaluated for 379 patients. The tumor tissue and normal tissue (normal pancreatic tissue or lymph nodes without cancer cells) were obtained through surgical resection.

### 2.2. DNA Isolation

The normal DNA was obtained from the whole blood (*n* = 363) or formalin-fixed paraffin-embedded (FFPE) normal tissues (*n* = 171). The tumor DNA samples were obtained from FFPE tumor tissues. All FFPE blocks were analyzed by a pathologist for verification and the selection of only tumor or normal cells for extraction. DNA was extracted from whole blood using DNeasy Blood and Tissue Kits (QIAGEN, Hilden, Germany). DNA from the FFPE tissues was isolated using a blackPREP FFPE DNA Kit (Analytik Jena, Jena, Germany) according to the manufacturer’s instructions. The quantity of nucleic acids was controlled using a Qubit fluorometer (Invitrogen, Life Technologies Corporation, Waltham, MA, USA). NGS and MSI testing were both performed on DNA obtained from the same extraction.

### 2.3. Library Preparation and Sequencing

DNA (100 ng for whole blood or 500 ng for FFPE tissue) was sheared to 200 bp using the Covaris S 220 System (Covaris, Woburn, MA, USA). It was then subjected to library preparation with the KAPA HyperPlus Kit (Roche, Rotkreuz, Switzerland) according to the manufacturer’s instructions. To analyze the *MLH1*, *MSH2*, and *MSH6* genes (all exons ± 20 bp in bordering introns), we designed a customized panel for targeted DNA sequencing using the NimbleDesign Software 4.3 (Roche). The NGS was performed on a NextSeq 500 System (Illumina, San Diego, CA, USA) under 76 × 2 bp paired-end mode and a MiniSeq 500 System (Illumina) under 151 × 2 bp paired-end mode. The average coverage was at least 500× for tumor tissues and at least 100× for blood and normal tissues.

### 2.4. Bioinformatics Analysis and Variant Characterization

Trimmed reads were mapped to the human genome (GRCh37) with BWA 0.7.17 [35]. Variant calling mainly relied on the GATK HaplotypeCaller 4.0.8.1 [36] but other tools were also used (FreeBayes 1.3.2 [37], Strelka 2.9.10 [38], and VarDict [39]). Variant filtering was then performed. Finally, all variants of interest were carefully inspected with an Integrative Genomic Viewer (IGV). Somatic mutations were identified using Mutect2 (GATK 4.0.8.1), taking into account FFPE artifacts either in paired mode (when matched normal tissue (or blood) was available), or in tumor-only mode. Details on the variant calling is presented in Supplementary Material S1.

The derived variant lists were annotated using ANNOVAR [40], including population frequency databases (gnomAD [41], 1000 Genomes, Kaviar, and others), conservation scores (PhastCons), amino-acid substitution-effect databases (SIFT, LRT, Polyphen2, FATHMM, MutationAssessor, MutationTaster, VEST3, PROVEAN, MetaSVM, M-CAP, MetaLR, MutPred, REVEL, DANN, and CADD). For further analysis, variants with a population frequency threshold > 0.5% were filtered out.

The classification of variants was conducted in accordance with the recommendations of the American College of Medical Genetics and Genomics and the Association for Molecular Pathology (ACMG-AMP) [42]. Variants were classified as “Benign” (B), “Likely Benign” (LB), “Variant of Uncertain Significance” (VUS), “Likely Pathogenic” (LP), or “Pathogenic” (P). The variants annotated in ClinVar (with a review status of at least two stars) were categorized according to the designated pathogenicity category provided by ClinVar. Other variants were interpreted using InterVar [43]. The variants not annotated in ClinVar and InterVar, which were high-impact variants (stop gain/loss, frameshift indels, or canonical splice site variants), were categorized as LP. VUSs were additionally subclassified using in silico suits (SIFT, LRT, Polyphen2, and other ones listed above). A variant was designated as “VUS_D” if at least 3/4 of these tools predicted deleterious effects; otherwise, it was classified as “VUS_ND”. The VUSs were additionally reviewed (the literature and databases) for final confirmation of the VUS_D or VUS_ND status. Additionally, P, LP, and VUS variants underwent manual verification using the IGV to ensure the exclusion of potential sequencing and analytical artifacts [44].

A variant was classified as germline if it was detected in normal tissue (or blood). A variant was classified as somatic if it was found in tumor tissue but not in the matched normal tissue (or blood). If only tumor tissue was examined, it was not possible to make an unambiguous conclusion about the germline or somatic status of the variant.

### 2.5. MSI Analysis

MSI analysis was performed using the five mononucleotide repeat loci (MNRs) (NR-27, NR-21, NR-24, BAT-25, and BAT-26) in a pentaplex PCR. The sequences of primers were previously described in [45]. The PCR mixture (final volume of 25 μL) consisted of 1× Hot Start Taq-DNA polymerase buffer; 3 mM MgCl_2_; 1 unit Hot Start Taq DNA polymerase (SibEnzyme, Nowosibirsk, Russia); 0.2 mM dNTPs; 0.04 µM of NR-27, NR-21, and NR-24 primers; 0.08 µM of BAT-25 primers; 0.07 µM of BAT-26 primers; and 30 ng DNA. The PCR cycling conditions were as follows: 3 min at 95 °C, followed by 40 cycles of 30 s at 95 °C, 30 s at 55 °C, 30 s at 72 °C, and then 72 °C for 6 min. PCR was performed using the T100 thermal cycler (Bio-Rad, Hercules, CA, USA). Fluorescently labeled products were analyzed by capillary electrophoresis using a 3500 Series Genetic Analyzer (Applied Biosystems, Thermo Fisher Scientific, Waltham, MA, USA). Allelic sizes for each marker were estimated using GeneMapper 5 software (Applied Biosystems, Thermo Fisher Scientific, USA).

If the tumor tissue demonstrated a deviation in microsatellite length for at least one locus, the tumor tissue was compared to normal tissue (or blood) from the same individual (if available). If nonidentical profiles were observed for zero, one, and ≥two mononucleotide loci, the tumor was categorized as microsatellite stable (MSS), MSI-low (MSI-L), and MSI-high (MSI-H), respectively.

## 3. Results

### 3.1. Patient Characteristics

A total of 620 patients who received medical care at the N.N. Blokhin Russian Cancer Research Center from 2000 to 2021 were included in the study. All patients had histologically confirmed adenocarcinoma of the pancreas. Of them, 340 patients (54.8%) were female (Table 1). The median age was 62 (ranging from 27 to 90). Most patients (522, 84.2%) had a locally advanced primary tumor (T3–T4). Regional lymph node metastases were found in 277 cases (44.7%). Distant metastases were revealed in 190 cases (30.6%). The predominant site of metastases was the liver (128 cases, 20.6%), followed by the lung (24 patients, 3.9%), and the peritoneum (23 patients, 3.7%). Ascites was presented in 16 patients (2.6%) at diagnosis.

A total of 266 patients (42.9%) had relatives with another cancer, including 237 patients (38.2%) with first-degree relatives having a cancer diagnosis. A personal history of another cancer was found in 20 patients (3.2%) from the evaluated cohort.

### 3.2. Sequencing

We evaluated the frequency of germline and somatic P/LP variants and VUSs in the *MLH1*, *MSH2*, and *MSH6* genes in patients with PC (Table 2). Germline P/LP variants were detected in 1.1% (6/534) of the patients, and VUSs were detected in 4.1% (22/534). Somatic P/LP variants and VUSs were identified in 2.6% (4/155) and 2.6% (4/155) of patients, respectively. Only tumor specimens were investigated in 86 patients, so the germline/somatic status of the identified mutations is unknown. P/LP variants were detected in two patients (2.3%, 2/86) but one of them simultaneously carried LP and P variants in the *MSH6* gene. VUSs were identified in two patients (2.3%, 2/86) but one of them simultaneously carried two VUSs in the *MSH6* gene. The whole list of identified P and LP variants and patient characteristics is indicated in Table 3. The full list of identified VUSs and patient characteristics is shown in Supplementary Table S1.

### 3.3. MSI Testing

FFPE tumor tissue from 241 PC patients was analyzed, and MSI status analysis was successful in 235/241 patients. None of the 235 patients had tumors classified as MSI-H. Notably, fourteen of them harbored germline (one with P/LP and five with VUS_D), somatic (four with P/LP and three with VUS_D), or variants with unknown germline/somatic status (one with VUS_D).

Approximately 3.0% (7/235) of PC samples had a length deviation for one of the loci (NR21 in five samples, NR24 in one, and BAT25 in one). For all of these samples, matched normal tissue (or blood) was available. We tested normal DNA and found full concordance in locus size between the tumor and the matched normal samples. Thus, the tumors in all patients in our study were MSS but 3% have variant alleles for one of the markers. The percentage of variant alleles was 0% for NR-27, 1.1% for NR-21, 0.2% for NR-24, 0.2% for BAT-25, and 0% for BAT-26.

## 4. Discussion

Germline mutations in the MMR genes are the underlying cause of Lynch syndrome (LS) [12,13,14]. People with LS are more likely to develop colorectal cancer (CRC) and also have an increased risk of other primary cancers, including PC [46]. Among patients with LS, patients with CRC are the most studied. When these patients underwent germline testing, mutations in the *MLH1* and *MSH2* genes were detected much more frequently (61–78%) than mutations in the *MSH6* and *PMS2* genes (21–39%) [47,48,49]. In primary CRC, germline mutations in MMR genes were detected in 24% of patients (8% in *MLH1*, 7% in *MSH6*, 5% in *MSH2*, and 4% in *PMS2*), while only 18% of tumors were MSI-H [50]. Recent data suggest that mutations in MMR genes are more common and less penetrant than previously thought. Win et al. modeled the carrier frequency of mutations in the four MMR genes for the general population: 0.051% for *MLH1*, 0.035% for *MSH2*, 0.132% for *MSH6*, and 0.140% for *PMS2* [51]. Interestingly, mutations in the *MSH6* and *PMS2* genes, which are more prevalent in the general population, are found to be relatively uncommon among patients with LS [52,53,54,55,56,57]. Apparently, germline mutations in the *MSH6* and *PMS2* genes are associated with a markedly lower cancer risk than in the *MLH1* and *MSH2* genes [58]. Although MMR mutations have been extensively studied in CRC, limited data are available regarding their presence in PC. In our study, germline P/LP variants in MMR genes were identified in 1.1% of unselected PC patients. The results are consistent with the frequencies obtained in other studies (0.5–1.4%) [59,60,61,62]. In our study, the P/LP variants in the *MLH1*, *MSH2*, and *MSH6* genes occurred with equal frequency at 0.4% (2/534), while germline VUS_D variants were detected more often in the *MSH6* gene (1.5%, 8/534), compared to the *MLH1* (0.2%, 1/534) and *MSH2* (0.6%, 3/534) genes. None of the patients with P/LP/VUS_D variants showed signs of MSI. Given the limited number of identified variants, further studies in a larger sample size are necessary to obtain more accurate data on the *MLH1*, *MSH2*, and *MSH6* mutation frequencies in PC.

For PC patients with deleterious germline variants, evaluating the status of the wild-type allele within the tumor has great significance in determining the functional impact of an inherited variant. The MMR-D phenotype in tumors is thought to result from inactivation of the remaining functional allele due to somatic mutations, loss of heterozygosity, or epigenetic silencing (“second hit”) [63,64,65]. Recent studies have shown that the MMR-D phenotype could also be due to somatic biallelic alterations in MMR genes [66]. In our study, tumor sequencing was performed for 241 patients. Of these, 155 had a matched normal specimen, so that the somatic status of the mutation could be determined. Somatic P/LP and VUS_D variants were identified in 2.6% (4/155) and 1.9% (3/155) of PCs, respectively, but none of them were found in tumors with deleterious germline variants. However, it is important to note that one patient—for whom only the tumor was sequenced—carried both P and LP variants simultaneously, with variant allele frequencies (VAF) of 0.57 and 0.25, respectively. Based on the VAF values, we can only assume that the P variant was germline and the LP variant was somatic. Unfortunately, the MSI analysis of this patient’s tumor sample failed. Our results are in line with those obtained by Singhi et al., who identified somatic mutations in about 1.2% of 3594 PCs [60]. More research has focused on the analysis of PC tumors with a confirmed MMR-D phenotype. For example, Humphris et al. analyzed four MMR-D PCs and found that all of them had somatic mutations in MMR genes [29]. In contrast, Maple et al. analyzed three MMR-D PCs and found that all of them harbored germline mutations. Connor et al. analyzed four MMR-D PCs, with one exhibiting a somatic mutation and three displaying germline mutations in MMR genes [67]. Consequently, an analysis of both germinal and somatic alterations is crucial for the determination of the causes underlying the development of the MMR-D phenotype.

In recent years, the evaluation of MMR-D as a biomarker for immunotherapy has gained importance for numerous different tumors; however, the existing data on the prevalence of MMR-D in PC are limited and generally discordant. The wide range of MMR-D frequency in PC (from 0% to 75%) is probably the result of using different detection methods or evaluation criteria and the study of various sample sizes and compositions [19,27,28,32,33,34,68,69,70,71,72,73,74]. MMR-D tumors can be identified through MSI testing [10]. MSI can be detected by PCR using a panel of several microsatellite markers. The first panel, recommended by the National Cancer Institute for MSI analysis—the “Bethesda panel”—consists of two MNRs (BAT25 and BAT26) and three dinucleotide repeat loci (D2S123, D17S250, and D5S346) [75]. As an alternative, a panel of five MNRs (BAT25, BAT26, NR21, NR24, and NR27) was developed later [76,77]. Using this panel in MSI testing resulted in a significantly higher reproducibility level than the Bethesda panel [77]. Furthermore, the panel of five MNRs is quasimonomorphic and does not require a matched normal DNA for MSI analysis [45,78,79]. In published studies, not only the above panels were used for MSI analysis in PC [10,19,27,31,34,68,69,74]. Differences in the number (from three to twelve), type of markers used, and variations in the MSI-H classification criteria could have led to the wide range of published MSI frequencies in PC. Older studies reported high MSI rates in PCs but these incidences may have been overestimated. For example, Brentnall and colleagues analyzed up to twelve different dinucleotide markers in PC samples and detected length deviation at least in one locus in 75% of tumors [27]. Many publications refer to the Brentnall study as confirmation that MSI was found in 75% of PC cases but do not consider the outdated evaluation criteria used in this publication. In our study, the panel of five MNRs was used. No MSI-H tumors were detected in our cohort of 235 consecutive cases of surgically resected PC. Similar results were obtained in the largest studies examining unselected groups of patients with PC using the same PCR panel: 0.3–0.8% [10,19]. It is worth noting that, although markers BAT25, BAT26, NR21, NR24, and NR27 are considered quasimonomorphic, their variant allele frequencies greatly differ between populations [45]. In our study, 3% of patients have variant alleles for one of the MNRs. The percentage of variant alleles was 0% for NR-27, 1.1% for NR-21, 0.2% for NR-24, 0.2% for BAT-25, and 0% for BAT-26. The obtained allele frequencies correspond to a European population [45].

Finally, we sought to ascertain the relationship between mutations in the *MLH1*, *MSH2*, and *MSH6* genes and the MSI status in PC. Although in our study none of 235 MSI-evaluable PCs were MSI-H, fourteen of them harbored germline (one with P/LP and five with VUS_D), somatic (four with P/LP and three with VUS_D), or variants with unknown germline/somatic status (one with VUS_D). These data indicate that for patients with proven LS, the evaluation of tumor MMR-D status is of fundamental importance when deciding whether to prescribe immunotherapy. The conclusions of our study are limited because the MMR-D status was tested only using MSI-PCR, whereas IHC was not performed. Theoretically, in these cases, IHC could identify a certain percentage of tumors with l-MMR. However, the literature data indicate that LS is not always associated with the MMR-D phenotype in many tumors. A large study from the Memorial Sloan Kettering Cancer Center analyzed 15,045 solid tumors, including pancreatic carcinomas. Of 103 patients with mutations in the Lynch genes, the MSS phenotype was detected in 37 (36%) cases [80]. In the study by Singhi et al., the targeted genomic profile analyses were performed for 3594 PC samples. The germline and/or somatic genomic alterations in MMR genes were detected in about 1.7% of cases but only 0.1% of PCs were MSI-H [60]. Humphris et al. identified four MMR-D tumors (from 385 PC cases), all of which exhibited distinct mechanisms of somatic inactivation of MLH1 and MSH2; however, the authors noted that in their cohort, germline variants did not contribute to the MMR deficiency even in those with familial PC or a personal or family history of Lynch-related tumors [29]. The reason for the absence of the MMR-D phenotype in pancreatic carcinomas in LS patients is not entirely apparent and requires further investigation. It may lie in the fact that PC carcinogenesis in these patients is not associated with an impaired MMR system.

Several other limitations of our study are worth mentioning. The *MLH1*, *MSH2*, and *MSH6* genes but not *PMS2* and *EPCAM,* were analyzed. Tumor tissue was available for somatic sequencing and MSI testing for only 241 of the 620 PC patients, and the matched normal and tumor samples were investigated only for 155 of the 241 patients. Unfortunately, not all patients had a thorough clinical description, including their personal and family history of malignancy.

## 5. Conclusions

In our study of an unselected cohort of PC patients, germline and somatic alterations in MMR genes were detected; however, they did not contribute to the MMR-D phenotype. Our findings underscore the importance of assessing tumor MMR-D status in PC patients with confirmed LS when deciding whether to prescribe immunotherapy.

## Figures and Tables

**Table 1:** Clinicopathological characteristics of the analyzed cohort.

Characteristic	Value (*n* = 620)
Female, *n* (%)	340 (54.8%)
Age in years, median (min–max)	62 (27–90)
T stage at diagnosis, *n* (%)	
T1–T2	76 (12.3%)
T3–T4	522 (84.2%)
Tx	22 (3.5%)
N status at diagnosis, *n* (%)	
N0	296 (47.7%)
N1	277 (44.7%)
Nx	47 (7.6%)
М status at diagnosis, *n* (%)	
M0	409 (66%)
M1	190 (30.6%)
Mx	21 (3.4%)
Primary tumor location in the head of the pancreas, *n* (%)	413 (66.6%)
Metastases in retroperitoneal lymph nodes at diagnosis, *n* (%)	27 (4.4%)
Liver metastases at diagnosis, *n* (%)	128 (20.6%)
Peritoneal metastases at diagnosis, *n* (%)	23 (3.7%)
Lung metastases at diagnosis, *n* (%)	24 (3.9%)
Ascites at diagnosis, *n* (%)	16 (2.6%)
Personal history of other cancer, *n* (%)	20 (3.2%)
Number of relatives with other cancer, *n* (%)	
0	261 (42.1%)
1–2	255 (41.1%)
≥3	11 (1.8%)
Not known	93 (15%)
Presence of first-degree relatives with ovarian, breast, prostate, or pancreatic cancer, *n* (%)	237 (38.2%)

**Table 2:** The frequency of germline and somatic P, LP, and VUS variants in *MLH1*, *MSH2*, and *MSH6* genes in patients with PC.

Pathogenicity Classification	*MLH1*	*MSH2*	*MSH6*
germline (534 patients)			
P/LP	2 (0.4%)	2 (0.4%)	2 (0.4%)
VUS_D	1 (0.2%)	3 (0.6%)	8 (1.5%)
VUS_ND	4 (0.7%)	2 (0.4%)	4 (0.7%)
somatic (155 patients)			
P/LP	0	2 (1.3%)	2 (1.3%)
VUS_D	0	2 (1.3%)	1 (0.6%)
VUS_ND	0	1 (0.6%)	0
somatic/germline status is unknown (86 patients)			
P/LP	0	0	2 (2.3%) patients but one of them harbored two (P and LP) variants
VUS_D	0	1 (1.2%)	0
VUS_ND	0	0	1 (1.2%) patient who carries two VUS_ND

Abbreviations: LP, likely pathogenic; P, pathogenic; VUS, variants of uncertain significance; VUS_D, VUS for which at least 3/4 of the in silico algorithms used in this study predict that it is deleterious; VUS_ND, VUS for which less than 3/4 of the in silico algorithms used in this study predict that it is deleterious.

**Table 3:** Characteristics of PC patients with germline and somatic P/LP variants in the *MLH1*, *MSH2,* and *MSH6* genes.

Case No.	Sex	Age	Other Personal History of Cancer (Age If Known)	Family History of Cancer (Relation, Age If Known)	Variant	Type	RS	Pathogenicity Classification	ClinVar Database	Normal Tissue (or Blood)	Tumor Tissue	Germline/ Somatic	MSI Status
1	m	50	No	SCC (mother, 73), GC (mat grandfather)	MLH1:NM_000249:c.1732_1743del: p.578_581del	in-frame_del	-	LP	-	Yes (VAF = 0.40)	NA	germline	NA
2	f	64	EC	CRC (father), LC (mother)	MLH1:NM_000249:c.588delA:p.K196fs	frameshift_del	rs63751653	P	P	Yes (VAF = 0.49)	NA	germline	NA
3	f	74	NA	NA	MSH2:NM_000251:c.1405delC: p.Leu469_Val470insTer	stopgain	rs1060502027	LP	P	No	Yes (VAF = 0.21)	somatic	MSS
4	f	63	NA	NA	MSH2:NM_000251:c.G2039A:p.R680Q	nsSNV	-	LP	-	No	Yes (VAF = 0.03)	somatic	MSS
5	m	67	No	BC (mother, >50)	MSH2:NM_000251:c.687delA:p.R230fs	frameshift_del	rs63749897	P	P	Yes (VAF = 0.36)	NA	germline	NA
6	f	66	BC (55, 64)	NA	MSH2:NM_000251:c.G1571A:p.R524H	nsSNV	rs63751207	LP	VUS	Yes (VAF = 0.53)	NA	germline	NA
7	f	69	NA	NA	MSH6:NM_000179:c.C2731T:p.R911Ter	stopgain	rs63751017	P	P	No	Yes (VAF = 0.06)	somatic	MSS
8	f	72	NA	NA	MSH6:NM_000179:c.C309A:p.Y103Ter	stopgain	-	LP	-	No	Yes (VAF = 0.24)	somatic	MSS
9	m	59	NA	NA	MSH6:NM_000179:c.A2906T:p.Y969F	nsSNV	rs63749919	LP	CIP: VUS(4); LB(1)	NA	Yes (VAF = 0.57)	NA	NA
					MSH6:NM_000179:c.C3013T:p.R1005Ter	stopgain	rs63750563	P	P	NA	Yes (VAF = 0.25)	NA	
10	f	45	NA	NA	MSH6:NM_000179:c.A2906T:p.Y969F	nsSNV	rs63749919	LP	CIP: VUS(4); LB(1)	Yes (VAF = 0.42)	Yes (VAF = 0.44)	germline	MSS
11	m	45	No	No	MSH6:NM_000179:c.C2314T:p.R772W	nsSNV	rs63750138	P	P	Yes (VAF = 0.46)	NA	germline	NA
12	m	62	NA	NA	MSH6:NM_000179:c.G4001A:p.R1334Q	nsSNV	rs267608122	P	P	NA	Yes (VAF = 0.26)	NA	NA

Abbreviations: BC, breast cancer; CIP, conflicting interpretation of pathogenicity; CRC, colorectal cancer; del, deletion; EC, endometrial cancer; f, female; GC, gastric cancer; LC, lung cancer; LB, likely benign; LP, likely pathogenic; m, male; mat, maternal; MSS, microsatellite stable; NA, not available; nsSNV, nonsynonymous single-nucleotide variant; P, pathogenic; PC, pancreatic cancer; SCC, sigmoid colon cancer; VAF, variant allele frequency; VUS, variants of uncertain significance.

## Data Availability

The data that support the findings of this study are available from the corresponding author upon reasonable request.

## Supplementary Materials

The following supporting information can be downloaded at: https://www.mdpi.com/article/10.3390/cancers16112111/s1, Table S1: Characteristics of PC patients with germline and somatic VUSs in the *MLH1*, *MSH2*, and *MSH6* genes. Supplementary Material S1: Bioinformatics analysis (in details). References [35,36,37,38,39,40,41,81] are cited in Supplementary Material S1.
