# Supplementary material for: Mutations in Mismatch Repair Genes and Microsatellite Instability Status in Pancreatic Cancer
Source: Cancers (Basel). 2024 May 31;16(11):2111. doi: 10.3390/cancers16112111 (PMC11171205; doi:10.3390/cancers16112111)
Supplement: Supplementary file 1 [file cancers-16-02111-s001.zip › Supplementary Material S1 31052024.pdf]

Supplementary Material S1.

Bioinformatics analysis (in details)

Illumina reads have been preprocessed with Trimmomatic 0.38 software (3′- and sliding window trimming, filtering, removal of any residual adapters). Next, the trimmed reads were mapped to the human genome (GRCh37) using BWA tool, version 0.7.17 [35]. All secondary (supplementary) alignments were removed with samtools (view -F 2048). BAM files were processed with Picard-tools 2.21.3 [81] and Samtools 1.10. Duplicated reads were identified with FixMateInformation and MarkDuplicatesWithMateCigar (Picard-tools).

The identification of variants was conducted mainly based on HaplotypeCaller (GATK 4.0.8.1) [36], but also other algorithms were used: FreeBayes 1.3.2 [37], Strelka 2.9.10 [38], VarDict [39]. This process was implemented in two distinct approaches: joint calling, which was conducted simultaneously across all samples, and single-sample calling. The analysis was restricted to specific genomic regions as outlined in the manifest file provided by Roche (with 50 bp padding). GATK HaplotypeCaller was started with enabled additional annotations (StrandBiasBySample, StrandOddsRatio, BaseQualityRankSumTest, MappingQualityRankSumTest, RMSMappingQuality, ReadPosRankSumTest, FisherStrand). VCFs generated with HaplotypeCaller were split into indels VCFs and single nucleotide variants (SNVs) VCFs and then transferred to GATK VariantFiltration tool. We filtered out SNVs with low confidence, strand bias, mapping quality, bias positional bias (QD < 2.0; QUAL < 35.0; MQ < 40; MQRankSum < -12.5; FS > 60.0; SOR > 3.0; ReadPosRankSum < -8.0). For indels, less stringent criteria were used (QD < 2.0; QUAL < 33.0; FS > 200.0; ReadPosRankSum < -20.0).

In majority of cases, we relied on the results from GATK HaplotypeCaller, but in addition Strelka, FreeBayes, VarDict were used. The default threshold parameters for these tools were adjusted to increase reliability depending on caller. Finally, we filtered mutations by Phred Quality score depending on the specific caller and situation in general.

Furthermore, for GATK and other callers, we marked suspectable substitutions in error-prone motifs (e.g., GGGTG > GGGGG, CCCG > CCCC). Finally, we excluded substitutions occurring simultaneously in a large number of samples with a variant allele frequency (VAF) below 20% (most possibly, mismappers or technical artifacts).

Nevertheless, the above filtering steps still resulted in the detection of a number of false substitutions, so all variants of interest were also carefully inspected manually with IGV.

Somatic mutations were identified using Mutect2 (GATK 4.0.8.1). When matched normal tissue (or blood samples) were available, Mutect2 was run in paired mode. If only tumor tissue was available, the analysis proceeded in "tumor-only" mode. We passed population frequency data from gnomAD 2.1.1 [41] to Mutect2. In order to eliminate FFPE artifacts, the LearnReadOrientationModel, GetPileupSummaries, and CalculateContamination tools (GATK) were used. Subsequently, the VCF files generated by Mutect2 underwent final filtration using the FilterMutectCalls tool (GATK). Additionally, paired tumor-normal samples underwent manual review to identify any variants that Mutect2 may have missed, ensuring a comprehensive analysis of somatic mutations.

The derived germline and somatic variant lists were annotated using ANNOVAR (June 2020 version) [40], including gnomAD [41], 1000 Genomes, Kaviar and other variant population frequency databases; conservation scores (PhastCons); Iand predicting the effect of amino acid substitutions (SIFT, LRT, Polyphen2, FATHMM, MutationAssessor, MutationTaster, VEST3, PROVEAN, MetaSVM, M-CAP, MetaLR, MutPred, REVEL, DANN, and CADD). For further analysis, variants with a population frequency threshold > 0.5% were filtered out.
