# Supplementary material for: Mutations in Mismatch Repair Genes and Microsatellite Instability Status in Pancreatic Cancer
Source: Cancers (Basel). 2024 May 31;16(11):2111. doi: 10.3390/cancers16112111 (PMC11171205; doi:10.3390/cancers16112111)
Supplement: Supplementary file 1 [file cancers-16-02111-s001.zip › Supplementary Table S1 31052024.pdf]

Supplementary table S1. Characteristics of PC patients with germline and somatic VUSs in the *MLH1*, *MSH2*, and *MSH6* genes

Case no.	Sex	Age	Other personal history of cancer (age if known)	Family history of cancer (relation, age if known)	Variant	Type	RS	Pathogenicity classification	ClinVar database	Normal tissue (or blood)	Tumor tissue	germline/ somatic	MSI status
13	f	72	NA	NA	MLH1:NM_000249:c.T1964C:p.I655T	nsSNV	rs63751225	VUS_ND	CIP: VUS(5); LB(6)	Yes (VAF=0.42)	Yes (VAF=0.46)	germline	MSS
14	m	31	No	No	MLH1:NM_000249:c.T1964C:p.I655T	nsSNV	rs63751225	VUS_ND	CIP: VUS(5); LB(6)	Yes (VAF=0.45)	NA	germline	NA
15	m	56	No	No	MLH1:NM_000249:c.T1612C:p.W538R	nsSNV	-	VUS_ND	-	Yes (VAF=0.50)	NA	germline	NA
16	f	61	CC	CRC (brother), CRC + PC (nephew)	MLH1:NM_000249:c.1611_1613del:p.W538del	in-frame_del	-	VUS_D	-	Yes (VAF=0.49)	NA	germline	NA
17	f	63	No	BC (mother), EsC (father)	MLH1:NM_000249:c.T1964C:p.I655T	nsSNV	rs63751225	VUS_ND	CIP: VUS(5); LB(6)	Yes (VAF=0.42)	NA	germline	NA
18	f	42	NA	NA	MSH2:NM_000251:c.C1826T:p.A609V	nsSNV	rs63750665	VUS_D	VUS	No	Yes (VAF=0.05)	somatic	MSS
19	m	46	NA	NA	MSH2:NM_000251:c.C1600T:p.R534C	nsSNV	rs63750029	VUS_D	CIP: VUS(7); LB(1)	No	Yes (VAF=0.08)	somatic	MSS
20	m	59	NA	NA	MSH2:NM_000251:c.A992G:p.N331S	nsSNV	rs779673318	VUS_D	CIP: VUS(4); LB(1)	Yes (VAF=0.58)	Yes (VAF=0.46)	germline	MSS
21	f	69	No	No	MSH2:NM_000251:c.G2422C:p.E808Q	nsSNV	-	VUS_ND	-	No	Yes (VAF=0.05)	somatic	MSS
22	f	65	No	PCa (father)	MSH2:NM_000251:c.G1681A:p.E561K	nsSNV	rs63750328	VUS_D	CIP: VUS(9); B(1)	NA	Yes (VAF=0.47)	NA	MSS
23	m	69	No	No	MSH2:NM_000251:c.G2178C:p.M726I	nsSNV	rs587782396	VUS_D	CIP: VUS(7); LB(1)	Yes (VAF=0.48)	NA	germline	NA
24	f	74	No	KC (father)	MSH2:NM_000251:c.G1217A:p.R406Q	nsSNV	rs146567853	VUS_ND	CIP: VUS(6); B(1); LB(6)	Yes (VAF=0.49)	NA	germline	NA
25	f	77	NA	NA	MSH2:NM_000251:c.A1430G:p.N477S	nsSNV	rs1558508152	VUS_ND	VUS	Yes (VAF=0.51)	NA	germline	NA
26	m	64	NA	NA	MSH2:NM_000251:c.G512T:p.R171M	nsSNV	-	VUS_D	-	Yes (VAF=0.51)	NA	germline	NA
27	f	70	No	EsC (mother), GC (sister)	MSH6:NM_000179:c.C3674T:p.T1225M	nsSNV	rs63750370	VUS_D	CIP: LP(1); VUS(7); LB(1)	Yes (VAF=0.46)	NA	germline	MSS
28	m	63	NA	NA	MSH6:NM_000179:c.C3737T:p.S1246L	nsSNV	-	VUS_D	-	No	Yes (VAF=0.28)	somatic	MSS
29	f	57	No	No	MSH6:NM_000179:c.C3674T:p.T1225M	nsSNV	rs63750370	VUS_D	CIP: LP(1); VUS(7); LB(1)	Yes (VAF=0.55)	Yes (VAF=0.27)	germline	MSS
30	m	56	No	No	MSH6:NM_000179:c.A3961G:p.R1321G	nsSNV	rs41295278	VUS_ND	CIP: VUS(11); LB(4)	Yes (VAF=0.47)	NA	germline	NA
31	f	59	NA	NA	MSH6:NM_000179:c.C1481T:p.A494V	nsSNV	rs1264762735	VUS_ND	CIP: VUS(2); LB(1)	NA	Yes (VAF=0.42)	NA	MSS
					MSH6:NM_000179:c.C968G:p.T323S	nsSNV	-	VUS_ND	-	NA	Yes (VAF=0.38)	NA	
32	m	64	NA	NA	MSH6:NM_000179:c.G2983A:p.E995K	nsSNV	rs63750258	VUS_D	VUS	Yes (VAF=0.54)	Yes (VAF=0.51)	germline	MSS
33	f	67	No	No	MSH6:NM_000179:c.13_14delAGinsTT:p.S5F	nsSNV	-	VUS_ND	-	Yes (VAF=0.55)	Yes (VAF=0.35)	germline	MSS
34	f	64	BC (50)	No	MSH6:NM_000179:c.T3758A:p.V1253E	nsSNV	rs202066386	VUS_D	CIP: VUS(9); LB(2)	Yes (VAF=0.50)	Yes (VAF=0.47)	germline	MSS
35	f	59	NA	NA	MSH6:NM_000179:c.T2384C:p.I795T	nsSNV	rs202127474	VUS_ND	CIP: VUS(6); LB(5)	Yes (VAF=0.51)	Yes (VAF=0.43)	germline	MSS
36	f	60	BC	GC (grandmother, 74)	MSH6:NM_000179:c.A1978G:p.K660E	nsSNV	-	VUS_D	VUS	Yes (VAF=0.56)	NA	germline	NA
37	m	49	No	BC (mother), KC (father)	MSH6:NM_000179:c.C2291A:p.T764N	nsSNV	rs561198849	VUS_ND	CIP: VUS(7); B(1)	Yes (VAF=0.48)	NA	germline	NA

Case no.	Sex	Age	Other personal history of cancer (age if known)	Family history of cancer (relation, age if known)	Variant	Type	RS	Pathogenicity classification	ClinVar database	Normal tissue (or blood)	Tumor tissue	germline/ somatic	MSI status
38	f	67	BC	No	MSH6:NM_000179:c.C3674T:p.T1225M	nsSNV	rs63750370	VUS_D	CIP: LP(1); VUS(7); LB(1)	Yes (VAF=0.51)	NA	germline	NA
39	f	61	NA	NA	MSH6:NM_000179:c.A2561T:p.K854M	nsSNV	rs34374438	VUS_D	CIP: VUS(5); B(1); LB(7)	Yes (VAF=0.46)	NA	germline	NA
40	f	68	NA	NA	MSH6:NM_000179:c.A1474G:p.M492V	nsSNV	rs61754783	VUS_D	CIP: VUS (7); LB(2)	Yes (VAF=0.44)	NA	germline	NA

Abbreviations: BC, breast cancer; B, benign; CC, cecum cancer; CIP, conflicting interpretation of pathogenicity; CRC, colorectal cancer; del, deletion; EsC, esophageal cancer; f, female; GC, gastric cancer; KC, kidney cancer; LB, likely benign; LP, likely pathogenic; m, male; MSS, microsatellite stable; NA, not available; nsSNV, nonsynonymous single-nucleotide variant; PC, pancreatic cancer; PCa, prostate cancer; VAF, variant allele frequency; VUS, variants of uncertain significance; VUS_D, VUS for which at least 3/4 of the *in silico* algorithms used in this study predict that it is deleterious; VUS_ND, VUS for which less than 3/4 of the *in silico* algorithms used in this study predict that it is deleterious.
